# Long-Term Neuroanatomical Consequences of Childhood Maltreatment: Reduced Amygdala Inhibition by Medial Prefrontal Cortex

**DOI:** 10.3389/fnsys.2020.00028

**Published:** 2020-06-03

**Authors:** Roman Kessler, Simon Schmitt, Torsten Sauder, Frederike Stein, Dilara Yüksel, Dominik Grotegerd, Udo Dannlowski, Tim Hahn, Astrid Dempfle, Jens Sommer, Olaf Steinsträter, Igor Nenadic, Tilo Kircher, Andreas Jansen

**Affiliations:** ^1^Department of Psychiatry and Psychotherapy, Department of Medicine, University of Marburg, Marburg, Germany; ^2^Centre for Mind, Brain and Behavior (CMBB), University of Marburg and Justus Liebig University Giessen, Marburg, Germany; ^3^Department of Neurology, Bayreuth Clinic, Klinikum Bayreuth GmbH, Bayreuth, Germany; ^4^Department of Psychiatry and Psychotherapy, University of Münster, Münster, Germany; ^5^Institute of Medical Informatics and Statistics, Kiel University, Kiel, Germany; ^6^Core-Unit Brainimaging, Faculty of Medicine, University of Marburg, Marburg, Germany

**Keywords:** major depression, childhood maltreatment, fMRI, connectivity, emotion processing, dynamic causal modeling

## Abstract

Similar to patients with Major depressive disorder (MDD), healthy subjects at risk for depression show hyperactivation of the amygdala as a response to negative emotional expressions. The medial prefrontal cortex is responsible for amygdala control. Analyzing a large cohort of healthy subjects, we aimed to delineate malfunction in amygdala regulation by the medial prefrontal cortex in subjects at increased risk for depression, i.e., with a family history of affective disorders or a personal history of childhood maltreatment. We included a total of 342 healthy subjects from the *FOR2107* cohort (www.for2107.de). An emotional face-matching task was used to identify the medial prefrontal cortex and right amygdala. Dynamic Causal Modeling (DCM) was conducted and neural coupling parameters were obtained for healthy controls with and without particular risk factors for depression. We assigned a *genetic risk* if subjects had a first-degree relative with an affective disorder and an *environmental risk* if subjects experienced childhood maltreatment. We then compared amygdala inhibition during emotion processing between groups. Amygdala inhibition by the medial prefrontal cortex was present in subjects without those two risk factors, as indicated by negative model parameter estimates. Having a *genetic risk* (i.e., a family history) did not result in changes in amygdala inhibition compared to *no risk* subjects. In contrast, childhood maltreatment as *environmental risk* has led to a significant reduction of amygdala inhibition by the medial prefrontal cortex. We propose a mechanistic explanation for the amygdala hyperactivity in subjects with particular risk for depression, in particular childhood maltreatment, caused by a malfunctioned amygdala downregulation via the medial prefrontal cortex. As childhood maltreatment is a major *environmental* *risk* factor for depression, we emphasize the importance of this potential early biomarker.

## Introduction

Major depressive disorder (MDD) is a common, chronic, costly, and debilitating disorder, affecting more than 300 million people worldwide (World Health Organization, 2017). The lifetime prevalence is in most countries in the range of 8–15% (Andrade et al., 2003; Kessler et al., 2003; Moffitt et al., 2010). MDD is caused by a complex interplay of genetic susceptibility and environmental factors, showing a heritability of ~35% (Otte et al., 2016). Genetic risk factors are believed to decrease resilience to environmental stressors and make disorder onset more probable. Environmental risk factors include stressful life events and, in particular, childhood maltreatment (Nelson et al., 2017). Childhood maltreatment leads to an increased risk for the development of recurrent MDD and a weaker response to treatment (Nanni et al., 2011). Childhood maltreatment is also associated with persistent neurobiological alterations in brain areas involved in mood regulation (Nemeroff, 2016), strongly resembling changes reported for MDD patients (Dannlowski et al., 2012). A deeper understanding how specific risk factors for depression alter the functional neuroanatomy is important not only from a basic neuroscience perspective, but also to identify neurobiological changes that might be used as biomarkers to potentially provide preventive measures to on-risk individuals at early stages.

Functional magnetic resonance imaging (fMRI) yielded insights into the neuroanatomical correlates of MDD. One robustly replicated finding is the hyper-responsiveness of the amygdala during emotion processing (e.g., Abler et al., 2007; Dannlowski et al., 2007; Siegle et al., 2007; Suslow et al., 2010; for meta-analysis, see Fitzgerald et al., 2008; Palmer et al., 2015). Changes in activity in the amygdala and accompanying changes of activity in the medial prefrontal cortex (mPFC) have led to the formulation of the *limbic-cortical model of major depression* (Graham et al., 2013). This model, first outlined by Mayberg and colleagues (Mayberg, 1997), considers MDD as a network disorder. One key aspect is that hyper-activity in limbic areas is not adequately controlled by prefrontal regions, with an associated depressed mood (Mayberg et al., 1999). More importantly, amygdala hyperactivity is also present in subjects at *genetic* (Joormann et al., 2012) and *environmental risk* for depression, such as childhood maltreatment (Dannlowski et al., 2012). This hyperactivity is therefore not specific for MDD but may indicate a general vulnerability to mental disorders.

The *limbic-cortical model* offers a testable framework that can continuously integrate neuroimaging findings with complementary neuroanatomical, neurochemical, and electrophysiological studies in the investigation of the pathogenesis of depression. In the following, we deliberately used a simplified version of the *limbic-cortical model of Major Depression*. Our model focuses on the connection between mPFC and amygdala. This allows, on the one hand, to test whether the mPFC down-regulates the amygdala during emotion processing, and on the other hand whether this downregulation is modulated by *risk* factors.

The present study had two aims. First, we tested the *limbic-cortical model* by assessing the strength of amygdala inhibition exerted by the mPFC during an emotion processing task in a large group of healthy subjects. Second, we tested whether *genetic* (i.e., familial) and *environmental risk* factors modulate amygdala inhibition. We operationalized those risks via a family history of affective disorders and childhood maltreatment, respectively. We hypothesized that both risk factors decrease the inhibitory influence of the mPFC on the amygdala (Frodl et al., 2010; van Harmelen et al., 2010; Dannlowski et al., 2012; Joormann et al., 2012). To investigate the inhibition of mPFC to the amygdala, we applied Dynamic Causal Modeling (DCM, Friston et al., 2003) for fMRI. DCM allows for inferences about the directionality of brain connectivity and aims at inferring neural interactions from observational data. As DCM is strongly hypothesis-driven, it allows us to test hypotheses within the borders of a network model. Furthermore, previous studies have used such models to decipher disorder and medication effects on limbic-cortical circuitry (de Almeida et al., 2009; Sladky et al., 2015a; Sladky et al., 2015b).

## Materials and Methods

### Subjects

Neuroimaging, clinical and neuropsychological data were obtained from the *FOR2107* cohort^1^. *FOR2107* is an ongoing multicenter study that aims to decipher the neurobiological foundations of affective disorders (Kircher et al., 2019). A detailed study description, including recruitment and assessment procedures, is given elsewhere (Vogelbacher et al., 2018; Kircher et al., 2019). Neuroimaging was performed at two centers, the University of Marburg and the University of Münster. The study was approved by the ethics committees of all participating institutions. Written informed consent was obtained from all subjects after a complete description of the study.

A first data freeze (v1.00) was conducted after 1,000 subjects (both patients and controls) were included in the study. For the selection of our final sample, we proceeded as follows: First, we decided to include only subjects measured at the University of Marburg to reduce variance related to different MR scanners (see Vogelbacher et al., 2018) for a comparison of data characteristics of both sites), leading to a sample size of 800 subjects. Second, we selected all subjects without any present or past psychiatric disorders, leading to a sample size of 352 subjects. Third, we excluded subjects with missing relevant imaging, clinical or neuropsychological data, leading to a final sample size of 342 (135 men, mean age 33.4 × 12.6 years, range 18–65 years). Subjects’ characteristics (sex, age, verbal IQ, years of education, BDI, and HAMD scores) are summarized in Supplementary Table S1.

The subjects were classified according to their risk status as having a *genetic risk* (i.e., familial risk, *n* = 63), an *environmental risk* (*n* = 44), or *no risk* factors (*n* = 247). Twelve subjects had both a *genetic* and *environmental risk*. G*enetic risk* was assigned if at least one first degree relative was suffering from an affective disorder. We use the word “genetic risk” as a proxy for a familial risk, knowing that we are not examining concrete genotypes (see “Discussion” section). An *environmental* *risk* was assigned when two subscales of the Childhood Trauma Questionnaire (CTQ, Bernstein et al., 1997) exceeded a critical threshold (10 for emotional abuse, eight for physical abuse, eight for sexual abuse, 15 for emotional neglect, eight for physical neglect). We hypothesized that both risk factors independently decreased the inhibitory influence of the mPFC on the amygdala (Dannlowski et al., 2012; Joormann et al., 2012).

### Experimental Design

All subjects were measured with a large neuroimaging battery assessing both brain function and structure. The study protocol is described in detail elsewhere (Kircher et al., 2019). In the present study, we analyzed the fMRI data from an emotional face-matching task (Hariri et al., 2002). It aims at activating face processing regions (e.g., fusiform face area, FFA), limbic regions (e.g., amygdala), and prefrontal regions. In the active condition, subjects viewed gray-scale images of fearful or angry faces (Ekman, 1992), in the control condition they viewed geometric shapes (circles and ellipsoids). In each trial, three items were presented. A target image was located at the top, two further images on the left and right side at the bottom, whereby one of these images was identical to the target image. The subject was instructed to indicate which of these two images was identical to the target image by pressing a corresponding button on an MRI-compatible response pad. The task was set up as block design, with six face and shape trials, respectively, per block. Blocks had a duration of 44 s (faces) and 32 s (shapes), respectively. Five shape blocks and four faces blocks were presented in an alternating order, starting with a shapes block. Blocks were separated by short inter-block-intervals. The paradigm lasted 6 min 14 s. Subjects of different subgroups performed similar with respect to hit rates and reaction times in this paradigm (Supplementary Table S2).

### MRI Data Acquisition

MRI data were acquired at a 3T MRI scanner (Tim Trio, Siemens, Erlangen, Germany), located at the Department of Psychiatry, University of Marburg, using a 12-channel head matrix Rx-coil. A T2*-weighted echo-planar imaging (EPI) sequence sensitive to blood oxygen level-dependent (BOLD) contrast was used with the following parameters: TE = 30 ms, TR = 2,000 ms, FoV = 210 mm, matrix = 64 × 64, slice thickness = 3.8 mm, distance factor = 10%, phase encoding direction anterior >> posterior, flip angle = 90°, no parallel imaging, bandwidth 2,232 Hz/Px, ascending acquisition, axial acquisition, 33 slices, slice alignment parallel to AC-PC line tilted 20° in the dorsal direction. A quality assurance (QA) protocol was implemented to monitor scanner stability by regular phantom measurements, similar to the “Glover protocol” implemented in the FBIRN consortium (Friedman and Glover, 2006). The QA protocol is described in detail elsewhere (Vogelbacher et al., 2018).

### MRI Data Analysis

#### Analysis of Brain Activity

fMRI data were analyzed with the software Statistical Parametric Mapping (SPM8, r2975)^2^ based on MATLAB 7.9.0 R2009b using standard routines and templates. *Preprocessing*: the initial three functional images were excluded from further analysis to exclude T1 stabilization effects. Functional images were realigned onto the mean image of the series using a six parameter rigid-body transformation, spatially normalized into standard MNI space, and resampled to a resolution of 2 × 2 × 2 mm^3^. Finally, the images were spatially smoothed using an 8 mm full-width-half-maximum (FWHM) isotropic Gaussian kernel. *Statistical analysis*: statistical analysis was performed using a general linear model (GLM) framework to create three-dimensional maps concerning the estimated regressor response amplitude. At the individual subject level, fMRI responses for both conditions (faces, shapes) were modeled in a block design using the canonical hemodynamic response function implemented in SPM8 convolved with a vector of onset times for the different stimulus blocks. High-pass filtering was applied with a cut-off frequency of 1/128 Hz to attenuate low-frequency components. Weighted beta-images and t-statistic images were created by contrasting the faces-condition (contrast weight 1) against the shapes-condition (contrast weight −1). At the group level, brain activation was assessed using a one-sample *t*-test for the contrast (faces > shapes).

#### Analysis of Brain Connectivity

Connectivity changes between the mPFC and the amygdala were assessed using Dynamic Causal Modeling [DCM, Friston et al., 2003), SPM12, r6685, DCM12, r6591]. DCM is a Bayesian framework for investigating the effective connectivity in a neural network based on neuroimaging data. In the present implementation, DCM describes the brain as a deterministic input-output system using a bilinear differential equation:

$$\frac{dz}{dt}=\left(A+\sum_{j=1}^{m}u_{j}B^{j}\right)z+Cu,$$

where *z* depicts the neuronal activities, *u* corresponds to the experimental input. *A* describes the endogenous (fixed or context-independent) connection strengths, *B^j^* defines how the experimental manipulation *u_j_* affects the connections among the network regions (modulatory connectivity), and *C* describes how the driving inputs directly influence the neuronal state of the network regions. The dynamics of the neuronal states in each region are translated into predictions of the measured BOLD signal by a hemodynamic forward model (Balloon-Windkessel model; (Buxton et al., 1998). Using a Variational Laplace approach with Gaussian assumptions on the prior and posterior distributions, the posterior densities of the model parameters (i.e., conditional mean and covariance) can be estimated by maximizing the negative free energy.

The starting point for a DCM analysis is the selection of a fixed set of regions, their possible connections, the driving inputs, and the modulatory inputs. Different models can be compared to identify which models best predict the data. DCM enables inferences at different levels, on the one hand, inference on model space, on the other hand, inference on parameter space of any given model. In the following, we will describe: (i) the extraction of time series in specific regions of interest (ROIs), the basis for estimating models; (ii) the model space definition; and (iii) the statistical inferences conducted with the model parameters of interest.

##### Time Series Extraction

**Figure 1:**
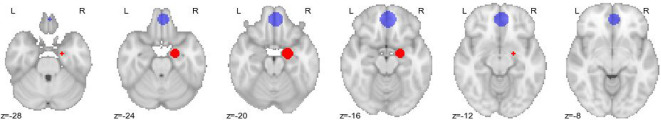
Graphical depiction of the regions-of-interest for the Dynamic Causal Modeling (DCM) analysis. Medial prefrontal cortex (mPFC; blue; peak voxel at MNI coordinates 2, 46, −16) and right amygdala (red; peak voxel at MNI coordinates 20, −6, −20) are shown on axial slices. As the center of the sphere, we used the peak voxels of the group-level activation map. Numbers indicate the MNI z-coordinate.

fMRI time series were extracted from the mPFC and the right amygdala, analogous to the procedure described by Sladky et al. (2015b). First, we calculated the group activation pattern for the contrast (faces > shapes) using a one-sample *t*-test on the weighted beta-images of all subjects. We determined mPFC (MNI: 2, 46, −16) and right amygdala (MNI: 20, −6, −20) by selecting voxels that showed the most significant activations concerning the *t*-test in those areas. Subsequently, we identified the single subject peak voxel coordinates using a searchlight approach. For this, single subjects’ activation maps were thresholded at *p* < 0.99, uncorrected, and the most strongly activated voxel was determined for each subject for the mPFC (within a search radius of 12 mm around group peak) and the right amygdala (within a search radius of 8 mm around group peak). See Figure 1 for a graphical depiction of the localization of the regions. We selected such a liberal threshold to avoid dropping single subjects due to sub-threshold activation out of our DCM analysis. This would have created a selective sample with only “strongly”-activating subjects and generalizations would not have been possible.

At last, the first principal component of the time series in the mPFC and the right amygdala, respectively, was extracted including all voxels inside a radius of 4 mm around the subject-specific peak voxel.

##### Model Space Definition

**Figure 2:**
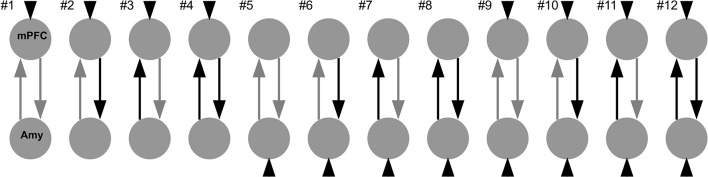
Model space consisting of 12 different DCMs. Faces with emotional expressions served as input into the system (C-matrix, short arrows), either on the mPFC, the amygdala, or both regions. The two regions were always reciprocally connected (A-matrix, grey arrows). Faces either modulated one connection, both connections, or none of the connections (B-matrix, black arrows).

Based on the *limbic-cortical model* of major depression (see “Introduction” section), we investigated the coupling between the mPFC and the right amygdala in a two-region model (Figure 2). We chose the right rather than bilateral amygdala because the most consistent findings regarding connectivity and risk factors focus on the right amygdala (e.g., Del-Ben et al., 2005; Anderson et al., 2007; Dalby et al., 2010; Windischberger et al., 2010; Dannlowski et al., 2012; Zhang et al., 2012; Sladky et al., 2015b). The choice of our model space was motivated by previous studies using a similar approach (de Almeida et al., 2009; Sladky et al., 2015a,b). We assumed reciprocal structural connectivity between both regions (Klingler and Gloor, 1960; Catani et al., 2002; Ghashghaei and Barbas, 2002). Therefore, the A-matrix was identical in all models. We created 12 different models, differing in their B- and C-matrices. The face blocks served as direct driving input (C-matrix) into the system, either via the mPFC, the amygdala, or both regions. These face regressors served also as modulatory input (B-matrix) on the connection from mPFC to the amygdala, on the connection from the amygdala to mPFC, on both connections or none connection.

##### Statistical Inference

We assessed the impact of risk status on amygdala inhibition. Our parameter of interest was, therefore, the modulatory B-matrix parameter of the fronto-amygdala connection. Bayesian Model Averaging (BMA) was conducted over the whole model space of a subject to compute a weighted average of each model parameter. The weighting was determined by the posterior probability of each model. This approach is considered as useful complementation to Bayesian Model Selection (BMS, Stephan et al., 2009) when none of the models tested outperformed all others (as was the case in the present study; see Supplementary Table S3).

A Bayesian estimation (*BEST*) procedure implemented in R (version 3.5.1; Kruschke, 2013) was used to calculate group differences. As input data, we used the posterior point estimates of all subjects’ DCM parameters (i.e., modulatory fronto-amygdala connection) after subject-specific BMA. We used uninformative default priors. In a first step, a Bayesian MCMC process generated random draws from the posterior distribution of group means and differences of means (500,000 samples each). We used the distribution of mean differences to infer the credibility of group differences. With this, posterior distributions for group mean comparisons were generated, similar to a *t*-test. But rather than *p*-values, Bayesian estimation provides probabilistic statements about values of interest (for more information, see Kruschke, 2010, 2013; Kruschke and Liddell, 2018). For example, we can state that with a probability of 95% the true value (i.e., mean connection strength) is higher for group A than for group B. Furthermore, an (e.g., 95%) highest density interval (HDI) marks a region of the credibility of parameter values. Obtaining a 95% HDI in the difference distribution that lies fully above or below zero, we can conclude a *credible difference*. Furthermore, we report effect sizes of the difference distribution between groups.

First, we computed three posterior distributions for the fronto-amygdala modulatory parameter, one for each group (*no risk*, *genetic risk*, and *environmental risk*). Subjects with *both risks* were included in both risk groups equally. We further computed the difference distributions between the respective risk groups and the *no-risk* group. We hypothesized that both risk factors independently decrease the inhibitory influence of the mPFC on the amygdala (Dannlowski et al., 2012; Joormann et al., 2012).

To account for confounding factors such as age, sex, and BDI score, we additionally conducted a multiple regression analysis (see Supplementary Analysis).

## Results

In the following, we will present subgroup-specific posterior parameter estimates after BMA and BEST. Our parameter of interest was the modulatory B-matrix parameter of the fronto-amygdala connection.

**Figure 3:**
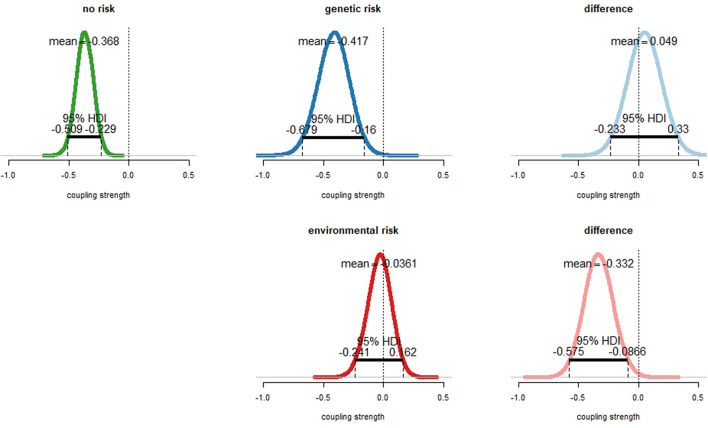
Effect of emotional face processing on the fronto-amygdala connection in healthy participants with and without particular risks for depression. Displayed are sampling distributions for the mean for each subgroup (left and middle column) as obtained via Bayesian estimation (“BEST”) and sampling distributions for the difference of group means (right column). Top row: subjects with *genetic risk* (family history) for major depressive disorder (MDD; top center) exhibited similar amygdala inhibition than those without risk (top left). 95% highest density intervals (HDIs) fell fully into the negative range. There was no credible difference between groups (top right). The 95% HDI well accumulated around zero. Bottom row: amygdala inhibition in healthy participants with *environmental risk* for depression (i.e., childhood maltreatment). Fronto-amygdala connectivity during emotional face processing was strongly diminished in healthy participants exhibiting an *environmental risk* with a probability of 99.5%, with the 95% HDI accumulating completely in the negative range.

For participants without any of our examined risk factors, the coupling between mPFC and amygdala was negative, characterized by a mean parameter estimate of −0.366 (Figure 3, top left). Importantly, the 95% HDI interval was completely below zero, indicating a credible difference from zero. In this group, the mPFC therefore clearly exerted an inhibitory influence on amygdala activity during face processing.

For participants with a family history of affective disorders (i.e., *genetic risk*), the coupling strength was similar (mean parameter estimate −0.417, Figure 3, top center). The 95% HDI was completely located in the negative range, indicating that also in this group the mPFC exerted a clear inhibitory influence on amygdala activity during face processing. The differences of means between the *no risk* and the *genetic risk* group were 0.049 (Figure 3, top right). Since both the distribution of differences between means accumulated at zero and the 95% HDI intersected zero, there was no evidence for a different coupling strength between both groups. The effect size of the difference distribution was 0.03 (Supplementary Figure S1).

For participants with an *environmental risk* (i.e., childhood maltreatment) the parameter estimate of the fronto-amygdala coupling accumulated around zero (mean parameter estimate −0.035, Figure 3, bottom center). The difference of means between the *no risk* and the *environmental risk* group was −0.331 (Figure 3, bottom right). Importantly, the mean of the *no-risk* group was with a probability of 99.5% more negative than the mean of the *environmental risk* group. Similarly, the 95% HDI was completely in the negative range (Figure 3, bottom right). This showed that the inhibitory influence of the mPFC on amygdala activity during face processing was diminished in the *environmental risk* group compared to the *no-risk* group. The corresponding effect size was −0.46 (Supplementary Figure S2).

An additional multiple regression analysis confirmed those results (see Supplementary Analysis). In the regression, we found an overall significant amygdala inhibition in subjects at *no risk* (*p* < 0.001), and a significant reduction of this inhibition by childhood maltreatment as *environmental risk* (*p* = 0.02, see Supplementary Analysis). Neither effects of age, sex, or BDI have been found.

## Discussion

In the present study, we tested a neurobiological model for the inhibition of the amygdala response to emotional stimuli in a large sample of healthy subjects. In particular, we tested whether this inhibition is modulated by *genetic* and *environmental risk* factors such as a family history of affective disorders and childhood maltreatment, respectively. Our results showed that amygdala inhibition by medial prefrontal cortex regions was strongly diminished in subjects who experienced childhood maltreatment, but not in subjects with genetic (i.e., familial) risk factors.

In the following, we will first discuss some background on the amygdala function and the necessity of amygdala inhibition. Then we will introduce the *limbic-cortical model* for depression. We will demonstrate how this network model explains amygdala hyperactivity in on-risk subjects, particularly those with past childhood maltreatment. Our results complement findings of amygdala hyperactivation in subjects with childhood maltreatment, and we propose a mechanistic model for how this hyperactivation may be caused.

### The Amygdala Prefrontal Pathway in Emotion Regulation

Amygdala’s activity is generally associated with the processing of emotionally salient stimuli, e.g., fearful facial expressions (Davis, 1992; Adolphs, 2002; Fitzgerald et al., 2006; Pessoa and Adolphs, 2011). The amygdala can respond to biologically relevant stimuli quickly (Méndez-Bértolo et al., 2016), allowing for a fast modulation of specialized cortical processing as well as behavioral, vegetative and endocrine reactions (LeDoux, 1998). Proper amygdala functioning was therefore of major advantage throughout vertebrate evolution. However, amygdala activity needs regulation, for instance after a stimulus has been evaluated as harmless. Such control is functionally related to the prefrontal cortex (Kim and Whalen, 2009; Agustín-Pavón et al., 2012), in particular to the orbitofrontal cortex (ORB), ventromedial prefrontal cortex (vmPFC) and anterior cingulate cortex (ACC; Mayberg, 1997; Mayberg et al., 1999; Etkin et al., 2011; Motzkin et al., 2015). Studies report overlapping functionalities of these three medial frontal regions (Etkin et al., 2011; Marusak et al., 2016). Lesions in medial prefrontal areas are associated with impaired down-regulation of fear and anxiety (Agustín-Pavón et al., 2012; Motzkin et al., 2015), implicating its role as an emotion control region. Additionally, metabolic alterations of those regulatory regions have been found for disorders such as MDD, which are accompanied by impaired emotion control abilities (Portella et al., 2011).

The amygdala has reciprocal anatomical connections to medial prefrontal regions, e.g., via the uncinate fasciculus (UF; Ebeling and von Cramon, 1992; Thiebaut de Schotten et al., 2012; Von Der Heide et al., 2013), which has been linked to inhibitory signaling from the mPFC to the amygdala (Kim and Whalen, 2009; Motzkin et al., 2015). Top-down signaling from mPFC towards the amygdala may be regarded as *safety signaling*, with the mPFC supposedly calming down the amygdala (Harrison et al., 2017). Dysfunctions of amygdala downregulation in MDD have been associated with structural abnormalities in the UF, showing, for instance, an inverse relationship between UF volume and trait anxiety (Kim and Whalen, 2009; Baur et al., 2012) and weakened UF white matter structural integrity in MDD (de Kwaasteniet et al., 2013), particularly right-hemispheric (Dalby et al., 2010; Zhang et al., 2012). In an often-used analogy, the amygdala is regarded as a barking watchdog, while the mPFC is the dog’s owner, evaluating the relevance of the barking dog and therefore differentiating between harmless and potentially hazardous events. In MDD however, the owner fails to regulate his or her watchdog as effectively as necessary, and the dog keeps alarming longer or louder as usual.

### The Limbic-Cortical Model

A network model describing the interaction of mPFC and amygdala was first outlined by *Mayberg and colleagues* in the context of MDD (Mayberg, 1997). Its initial formulation proposed aberrant networking of a variety of cortical and subcortical areas. It proposes hypo-activity in the dorsal cortical and dorsal limbic areas and accompanying hyperactivity in ventral (para-) limbic areas in MDD. This activation pattern was supposed to flip with treatment (Mayberg, 1997), and medial prefrontal areas are to mediate between those major compartments (Mayberg, 1997). It’s baseline activity has further been proposed as a biomarker for treatment success (Mayberg, 1997). Over the years the Mayberg model has been adapted and revised in very different fashions. For instance, the ventromedial prefrontal cortex (vmPFC) is often described as the regulatory region, inhibiting the amygdala in healthy subjects (e.g., Johnstone et al., 2007; Dutcher and Creswell, 2018) and lacking such inhibition in MDD (e.g., Johnstone et al., 2007). Other studies assigned such a regulatory function rather than the (ORB, Sladky et al., 2015b), but also (ACC, Johnstone et al., 2007; Etkin et al., 2011). In neuroimaging studies, regions such as vmPFC, ORB, and sometimes ACC are named in a very heterogeneous fashion, complicating the comparison of studies and findings. We derived both regions of interest from local peaks within the respective areas. Therefore, we named our prefrontal region, which encompassed both vmPFC and medial ORB, “mPFC” to keep it sufficiently general.

We applied the *limbic-cortical* *model* to data derived by healthy subjects with and without particular risk status for MDD rather than MDD patients themselves. We hypothesized that both of our examined risks may be associated with aberrant networking of this emotion regulation circuit, which then, in turn, may contribute to disorder onset. In the present study, we are not able to evaluate a causality chain due to the cross-sectional data used. However, we were able to evaluate the network model in healthy individuals without those two risk factors by showing, that there is indeed a down-regulation of the amygdala by mPFC during emotion processing, indicated by negative parameter estimates. We then examined how the network model behaves in subjects at-risk. In future studies, using longitudinal data that is currently collected in the *FOR2107* cohort, we will be able to further refine our findings by applying our models also to patient data.

### The Impact of Risk Factors

MDD is most likely caused by a combination of some polygenetic predisposition and environmental factors. Showing high heritability, a family history of MDD may have a major impact on an individual, e.g., lowering resilience to adverse life events (Joormann et al., 2012). On the other hand, there are environmental factors, elevating the probability of clinical depression. One factor, leading to increased risk for depression, is childhood maltreatment (Kessler, 1997; Gilbert et al., 2009). Childhood maltreatment probably leads to psychological and biological vulnerabilities and higher sensibility to stressors (Kessler, 1997; Beck, 2008; Danese et al., 2008; Nanni et al., 2011), increasing the probability of disorder onset. Furthermore, MDD patients that experienced childhood maltreatment show lower treatment outcome (Hammen et al., 2000; Lanquillon et al., 2000; Nanni et al., 2011). On a neural system level, healthy subjects with a family history of MDD show amygdala hyperactivity in emotional tasks (Joormann et al., 2012). Similarly, healthy subjects with childhood trauma experiences show amygdala hyperactivity as a response to emotional faces, much like patients suffering from MDD (Dannlowski et al., 2012), accompanied with structural alterations in the prefrontal cortex (Frodl et al., 2010; van Harmelen et al., 2010; Dannlowski et al., 2012). Early life events, therefore, may establish long-lasting changes in emotional processing and associated unfavorable alterations in brain structure, function, and connectivity.

In our analysis, we tackled the question of amygdala inhibition by mPFC in healthy subjects at-risk. We operationalized a *genetic risk* by assigning it to a subject if a first-degree relative ever had a diagnosed affective disorder. We found no credible differences in amygdala inhibition between the *no risk* and the *genetic risk* group (Figure 3, bottom). This was contrary to our hypothesis as we expected a weaker inhibition in those subjects under *genetic* (i.e., familial)* risk*. Likewise, the *environmental risk* was operationalized via childhood maltreatment (see “Materials and Methods” section). We found that childhood maltreatment was associated with a strong reduction of amygdala inhibition (Figure 3, bottom). In the framework of our network model—an operationalization of the *limbic-cortical model—*we, therefore, provide a mechanistic explanation for the observed amygdala hyperactivity in healthy subjects with childhood trauma experiences (Dannlowski et al., 2012), namely a failure of amygdala regulation by prefrontal control regions.

### Limitations

We acknowledge some limitations of our analyses. First, we used a simplified model including only two regions, covering only a small part of the brain regions associated with emotion processing. A widely distributed network of regions would form a better picture but comes with higher computational costs. Second, we identified one possible prefrontal region for our analysis, derived from our group activation data. Literature, however, reveals many different localizations of potential prefrontal control regions, with overlapping functionality but variability in their designations (Etkin et al., 2011; Marusak et al., 2016). We refer to the Mayberg studies with our results, which can be seen as the basis for the *limbic-cortical model* of MDD (Graham et al., 2013). It provides us a suitable framework for our hypotheses. However, the prefrontal control region we used differed from the regions within the original studies. Additionally, we operationalized a *genetic risk* via a family history of affective disorders. However, this does not capture any concrete genotype. With this kind of operationalization, we may also not distinguish between a true* genetic risk* due to inheritance, and an *environmental* factor such as emotional neglect due to the indirect consequences of a parent’s disorder. Therefore, our assigned *genetic risk* can be better understood as a familial risk, including both genetic and environmental factors.

### Conclusion

In this article, we constructed and evaluated a model proposing that childhood maltreatment but not a family history of affective disorders are characterized by a reduced inhibition of the amygdala by mPFC. In the context of our model, we illustrate a potential mechanism for the frequently reported amygdala hyperactivation in MDD during emotion processing. More importantly, the model provides a mechanistic explanation for amygdala hyperactivation in healthy subjects with childhood trauma experiences. Model parameters such as this may constitute vulnerability markers for clinical symptoms in later life and maybe predictive for treatment success. Information of such model parameters may be used for early therapeutic intervention in at-risk individuals, to prevent disorder onset and poor treatment response in later life stages, when pathological connections are tightened and more difficult to treat.

## Data Availability Statement

All PIs take responsibility for the integrity of the respective study data and their components. All authors and co-authors had full access to all study data. Code for crucial analyses as well as statistical maps, subject-specific DCM models, and further data is available in a public repository of the first author (https://github.com/kesslerr/limbiccortical).

## Ethics Statement

The studies involving human participants were reviewed and approved by Ethikkomission FB 20 Medizin, Baldingerstraße, 35032 Marburg & Ethik-Kommission der Ärztekammer Westfalen-Lippe und der Westfälischen Wilhelms-Universität Münster, Gartenstraße 210-214, 48147 Münster. The patients/participants provided their written informed consent to participate in this study.

## Author Contributions

RK: conceptualization of analyses, conduction of analyses, interpretation of the data, drafting, and revision of the manuscript. SS and TS: data collection, revision of the manuscript, and interpretation of the data. FS and DY: data collection. DG: data collection and provided data infrastructure. UD: design of fMRI protocol and financially enabled the study. TH: financially enabled the study and interpretation of the data, and revision of the manuscript. AD: financially enabled the study. JS and OS: provided data infrastructure. IN: financially enabled the study, and interpretation of the data. TK: design of fMRI protocol, financially enabled the study, and revision of the manuscript. AJ: conceptualization of analyses, conduction of analyses, interpretation of the data, provided data infrastructure, design of fMRI protocol, drafting and revision of the manuscript, and financially enabled the study.

## Conflict of Interest

TK received unrestricted educational grants from Servier, Janssen, Recordati, Aristo, Otsuka, neuraxpharm. Markus Wöhr is scientific advisor of Avisoft Bioacoustics. The remaining authors declare that the research was conducted in the absence of any commercial or financial relationships that could be construed as a potential conflict of interest.

## Footnotes

^1^www.for2107.de

^2^http://www.fil.ion.ucl.ac.uk/spm/

## Supplementary Material

The corresponding single-subject parameter estimates are displayed in Supplementary Figure S3. The Supplementary Material for this article can be found online at: https://www.frontiersin.org/articles/10.3389/fnsys.2020.00028/full#supplementary-material.
