# Supplementary material for: Long-Term Neuroanatomical Consequences of Childhood Maltreatment: Reduced Amygdala Inhibition by Medial Prefrontal Cortex
Source: Front Syst Neurosci. 2020 Jun 3;14:28. doi: 10.3389/fnsys.2020.00028 (PMC7283497; doi:10.3389/fnsys.2020.00028)
Supplement: Supplementary file 1 [file Data_Sheet_1.pdf]

Supplementary Material

1 Supplementary Methods

1.1 Subjects Characteristics

Table S1: Subjects' characteristics. N: Total number of subjects in the subgroup. HAMD is the Hamilton depression score (Hamilton, 1960). BDI is Becks Depression Inventory score (Beck, Ward, Mendelson, Mock, & Erbaugh, 1961).

group	no risk	genetic risk	environmental risk	both risks
**N**	247	51	32	12
**sex (m/f)**	99/148	20/31	13/19	3/9
**age**	33 ± 13	32 ± 13	40 ± 12	38 ± 13
**verbal IQ**	115 ± 14	114 ± 15	117 ± 16	113 ± 11
**years education**	14 ± 2	14 ± 3	14 ± 3	14 ± 3
**BDI**	4.2 ± 3.9	4.9 ± 5.6	5.4 ± 5.5	10 ± 7.7
**HAMD**	1.0 ± 1.5	1.6 ± 1.9	2.2 ± 2.5	4.0 ± 6.1

1.2 Task Performance

Table S2: Hit rates and reaction times (RT) for the different subgroups.

	mean hit rate faces	sd hit rate faces	mean hit rate shapes	sd hit rate shapes	mean RT faces	sd RT faces	mean RT shapes	sd RT shapes
**no risk**	0.91	0.25	0.89	0.25	1223.05	377.33	1041.71	295.47
**genetic risk**	0.90	0.26	0.88	0.26	1171.51	399.16	1005.99	306.22
**environmental risk**	0.91	0.25	0.90	0.24	1206.05	387.28	1030.48	304.58
**both risks**	0.91	0.25	0.90	0.24	1206.05	387.28	1030.48	304.58

1.3 Bayesian Model Selection

Table S3: Model exceedance probabilities and posterior probabilities. Bayesian Model Selection was conducted in each subgroup separately. Model exceedance probabilities describe the probability that one model is more like than all competing models generating the data. Posterior model probabilities determine the relative probability of a model and further its contribution of a model to the respective subgroups average model (BMA). For a graphical description of the twelve models see Figure 2. The subgroup-specific model probabilities as displayed here were not of particular importance for the group comparisons using BEST. Instead, we used subject-specific model probabilities to calculate each subject's individual average model.

**posterior probabilities**	**model**											
	1	2	3	4	5	6	7	8	9	10	11	12
**no risk**	0.01	0.01	0.01	0.30	0.02	0.02	0.04	0.36	0.00	0.04	0.15	0.04
**genetic risk**	0.02	0.03	0.02	0.16	0.02	0.04	0.03	0.30	0.02	0.10	0.18	0.09
**environmental risk**	0.04	0.11	0.08	0.18	0.03	0.05	0.04	0.29	0.03	0.07	0.04	0.04
**both risks**	0.04	0.04	0.05	0.22	0.05	0.06	0.09	0.20	0.05	0.09	0.05	0.07

**exceedance probabilities**	**model**											
	1	2	3	4	5	6	7	8	9	10	11	12
**no risk**	0.00	0.00	0.00	0.19	0.00	0.00	0.00	0.81	0.00	0.00	0.00	0.00
**genetic risk**	0.00	0.00	0.00	0.06	0.00	0.00	0.00	0.83	0.00	0.01	0.09	0.00
**environmental risk**	0.00	0.04	0.02	0.19	0.00	0.01	0.00	0.73	0.00	0.00	0.00	0.00
**both risks**	0.01	0.01	0.01	0.44	0.01	0.02	0.06	0.34	0.01	0.05	0.01	0.03

2 Supplementary Results

2.1 Bayesian Estimation

For both comparisons (either no risk vs. genetic risk or no risk vs. environmental risk) we used Bayesian Estimation ("BEST", (Kruschke, 2013)) to calculate e.g. differences of means. Furthermore, effect sizes, and differences in variances etc. are displayed in the following graphics.

2.1.1 No risk vs. genetic risk

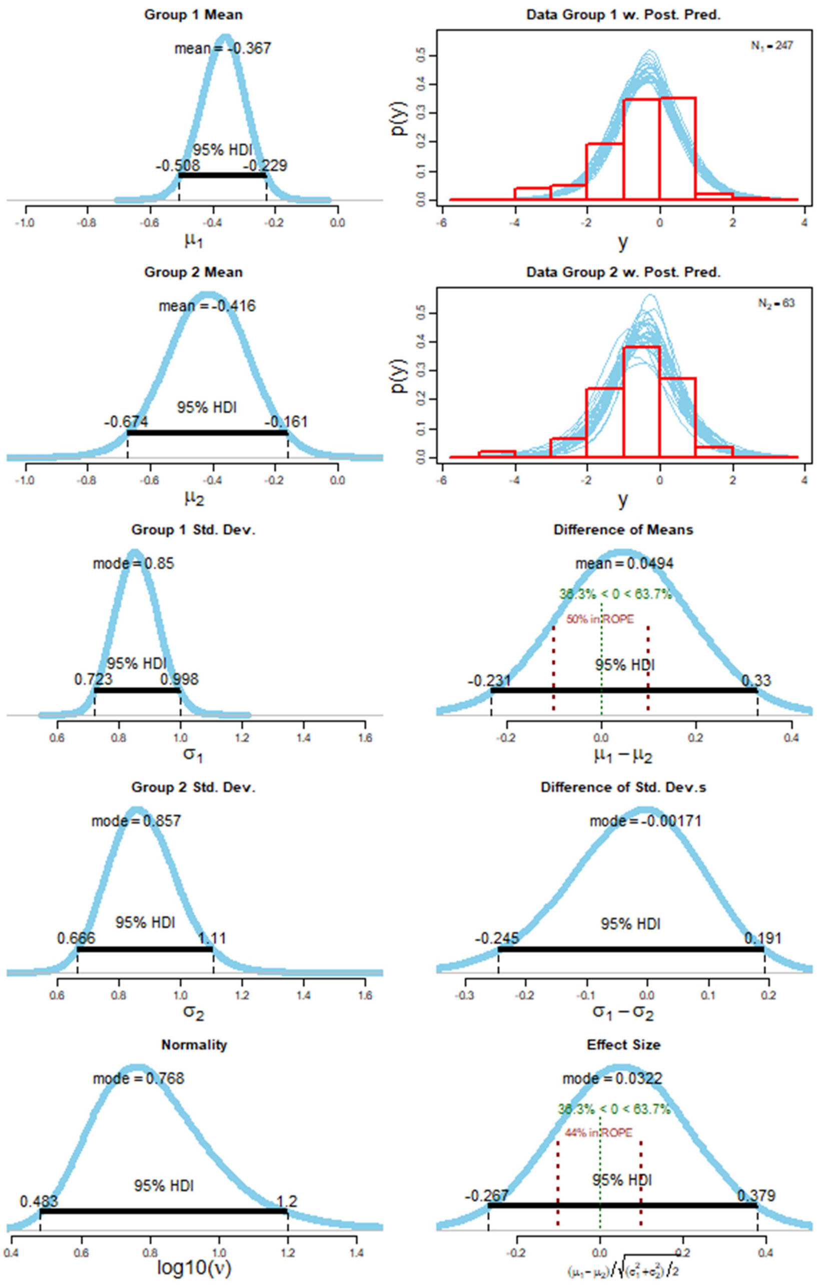

Figure S1: Detailed Bayesian Estimation ('BEST') results for the comparisons of subjects without risk (group 1) and subjects with genetic risk (group 2). Displayed are estimated group means and standard deviations, differences of means and standard deviations, degree of normality and effect sizes (Kruschke, 2013) alongside with highest density intervals (HDI).

2.1.2 No risk vs. environmental risk

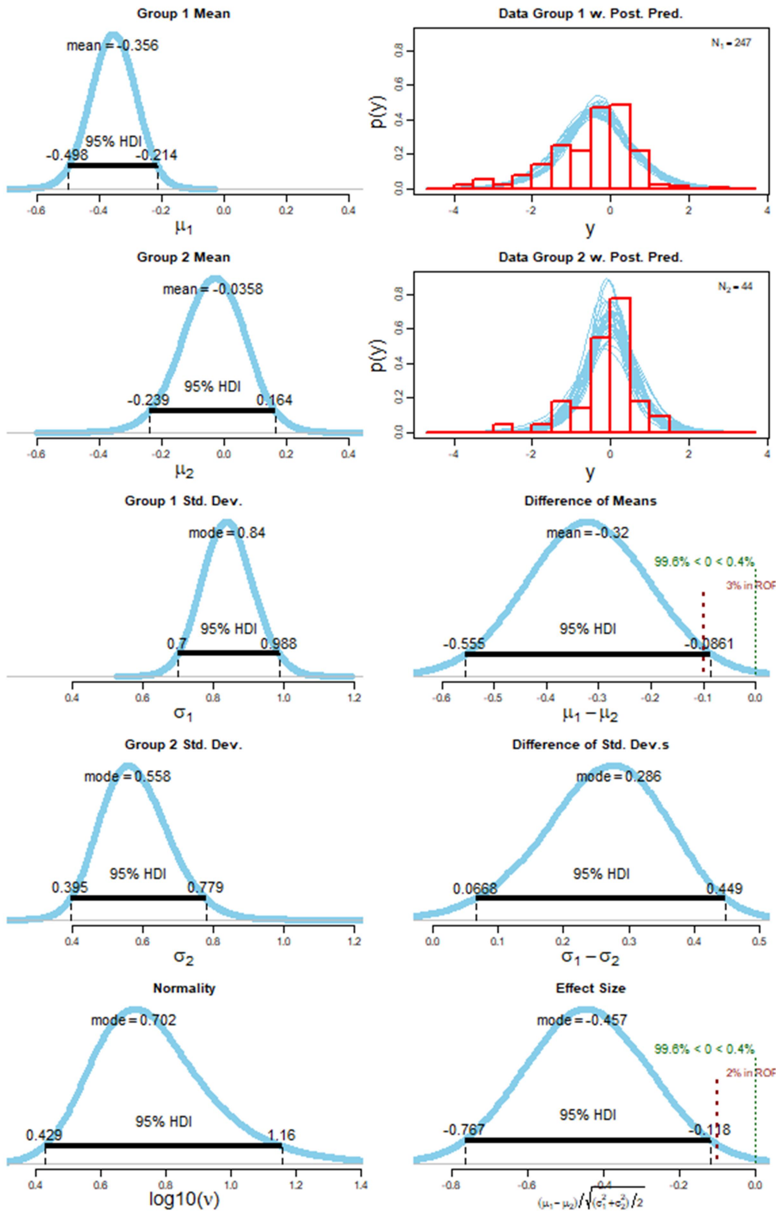

Figure S2: Detailed Bayesian Estimation ('BEST') results for the comparisons of subjects without risk (group 1) and subjects with environmental risk (group 2). Displayed are estimated group means and standard deviations, differences of means and standard deviations, degree of normality and effect sizes (Kruschke, 2013) alongside with highest density intervals (HDI).

3 Supplementary Analysis: Linear regression

Groups were not explicitly matched for sex, age, verbal IQ or years of education. Instead, we selected as many subjects as possible to increase the sensitivity of our analyses. To strengthen our results and to correct for these covariates, we additionally conducted a multiple linear regression analysis. We included covariates as age, sex, and BDI into our analysis as covariates.

3.1 Methods

Group differences between the modulatory B-matrix parameter of the fronto-amygdala connection were assessed using a linear regression model using R (version 3.5.1). We constructed the linear regression model of amygdala inhibition as a function of risk factors. As risk factors, we categorically modeled a family history (*genetic risk*) and childhood maltreatment (*environmental risk*). We further included major possible confounding variables as age (mean-centered), sex (female = 0, male = 1), and BDI in our model. The intercept of the model represents the average amygdala inhibition (if negative) of (female) subjects with none of the above risks and a BDI of zero. Other regression parameters give insight about the significance and strength of risk factors and confounding variables. If slopes are positive, the modeled factor decreases amygdala inhibition by mPFC. If negative, those factors increase amygdala inhibition. We further used a step-wise backward regression, by iteratively pruning the model parameters with highest p-value (until all $p<0.05$) to get a simpler model with only significant predictor variables.

3.2 Results

A multiple linear regression model was constructed to predict the influence of mPFC onto the amygdala during emotion processing. Predictors were *genetic risk* (family history, categorical), *environmental risk* (childhood maltreatment, categorical), age (mean-centered), sex (female = 0), and BDI. After stepwise backward regression using ordinary least squares (OLS), a significant regression equation was found ($p=0.02$) with an adjusted R-squared of 0.013. The predicted influence of mPFC onto the amygdala was -0.503 (intercept, 95% CI -0.618 & -0.387, $p<0.001$), with an increase of the parameter estimate by childhood maltreatment (binary) of 0.381 (95% CI 0.06 & 0.703, $p=0.02$). Therefore, the parameter estimate for healthy subjects at no risk was negative (i.e. amygdala inhibition). With childhood maltreatment, this inhibition was decreased significantly (i.e. reduced inhibition).

Using a full model without backward regression, we obtain similar results. Only the intercept (amygdala inhibition at no risk) and childhood maltreatment as predictor became significant. A family history, as well as covariates such as age, sex, and BDI, remained non-significant. The corresponding single-subject parameter estimates are displayed in Figure S3.

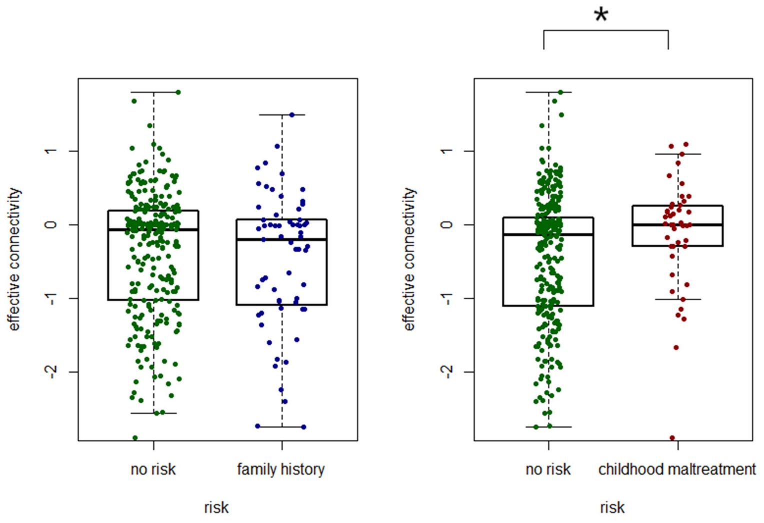

Figure S3: Single subject parameter estimates of the fronto-amygdala connection and result of multiple linear regression analysis.

4 Supplementary Analysis: Full-factorial analysis

To evaluate activation differences between groups, we constructed a 2x2 full-factorial analysis on the second level fMRI data. The first factor was *environmental risk* (childhood maltreatment), and the second factor *genetic risk* (family history).). We then analyzed "main effect of environmental risk" and "main effect of genetic risk" as F-contrasts. We masked the resulting contrast with the very same masks for right amygdala ("rAmy") and medial prefrontal cortex ("mPFC"), which we used to extract the time series for our subjects. No voxels exceeded significance in the F-contrasts at thresholds of $p<0.01$ (uncorrected for multiple comparisons). We therefore conclude no meaningful activation differences between no-risk and at-risk groups on activation level within our sample.

5 Supplementary References

Beck, A. T., Ward, C. H., Mendelson, M., Mock, J., & Erbaugh, J. (1961). Inventory for Measuring Depression. *Archives of General Psychiatry*, 561–571. https://doi.org/10.1001/archpsyc.1961.01710120031004

Hamilton, M. (1960). Scale for depression. *J. Neurol. Neurosurg. Psychiat.*, (23), 56–62.

Kruschke, J. K. (2013). Bayesian estimation supersedes the T test. *Journal of Experimental Psychology: General*, *142*(2), 573–588. https://doi.org/10.1037/a0029177
